# Development of a Reverse Genetic System to Generate Recombinant Chimeric Tacaribe Virus that Expresses Junín Virus Glycoproteins

**DOI:** 10.3390/pathogens9110948

**Published:** 2020-11-13

**Authors:** Sabrina Foscaldi, María Eugenia Loureiro, Claudia Sepúlveda, Carlos Palacios, María Belén Forlenza, Nora López

**Affiliations:** 1 Centro de Virología Animal (CEVAN), Consejo Nacional de Investigaciones Científicas y Técnicas (CONICET), Buenos Aires C1428EGA, Argentina; sfoscaldi@leloir.org.ar (S.F.); eugenialoureiro@yahoo.com.ar (M.E.L.); mbelenforlenza@gmail.com (M.B.F.); 2 Laboratorio de Virología, Departamento de Química Biológica, Facultad de Ciencias Exactas y Naturales, Universidad de Buenos Aires C1428EGA, Argentina; claudia@qb.fcen.uba.ar; 3 Instituto de Química Biológica de la Facultad de Ciencias Exactas y Naturales (IQUIBICEN), CONICET- Universidad de Buenos Aires, Buenos Aires C1428EGA, Argentina; 4 Instituto de Ciencia y Tecnología Dr. César Milstein (CONICET-Fundación Pablo Cassará), Buenos Aires C1440FFX, Argentina; cpalacios@fundacioncassara.org.ar

**Keywords:** Tacaribe virus, infectious clone, chimeric virus, Junín virus, non-coding region, viral attenuation

## Abstract

Mammarenaviruses are enveloped and segmented negative-stranded RNA viruses that comprise several pathogenic members associated with severe human hemorrhagic fevers. Tacaribe virus (TCRV) is the prototype for the New World group of mammarenaviruses and is not only naturally attenuated but also phylogenetically and antigenically related to all South American pathogenic mammarenaviruses, particularly the Junín virus (JUNV), which is the etiological agent of Argentinian hemorrhagic fever (AHF). Moreover, since TCRV protects guinea pigs and non-human primates from lethal challenges with pathogenic strains of JUNV, it has already been considered as a potential live-attenuated virus vaccine candidate against AHF. Here, we report the development of a reverse genetic system that relies on T7 polymerase-driven intracellular expression of the complementary copy (antigenome) of both viral S and L RNA segments. Using this approach, we successfully recovered recombinant TCRV (rTCRV) that displayed growth properties resembling those of authentic TCRV. We also generated a chimeric recombinant TCRV expressing the JUNV glycoproteins, which propagated similarly to wild-type rTCRV. Moreover, a controlled modification within the S RNA 5′ non-coding terminal sequence diminished rTCRV propagation in a cell-type dependent manner, giving rise to new perspectives where the incorporation of additional attenuation markers could contribute to develop safe rTCRV-based vaccines against pathogenic mammarenaviruses.

## 1. Introduction

*Arenaviridae* family members are enveloped viruses classified into four genera: *Hartmanivirus* and *Reptarenavirus* (reptilian), *Antennavirus* (discovered in fish), and *Mammarenavirus*, including viruses associated with rodent hosts, with the unique exception of Tacaribe virus (TCRV) [1]. TCRV was first isolated from bats (*Artibeus jamaicensis* and *Artibeus lituratus*) [2] and more recently from lone star ticks (*Amblyomma americanum*) [3]. However, the role of *A. americanum* in the ecology of the virus is unknown and experimental evidence obtained with the only remaining strain of TCRV (TRVL-11573) suggests that *A. jamaicensis* bats may not be a reservoir host for TCRV [4]. Mammarenaviruses are divided into New World (NW) and Old World (OW) complexes, according to phylogenetic and antigenic features and geographical distribution. The OW group includes Lassa virus (LASV), the causative agent of Lassa fever, a human hemorrhagic disease endemic to Western Africa. Within the NW group, viruses are subdivided into four clades: A, B, C, and A/Rec (Clade D). The prototypic TCRV is classified into Clade B along with all known human-pathogenic viruses that produce hemorrhagic disease in South America: Junín (JUNV), the etiological agent of the Argentinian hemorrhagic fever (AHF), and Machupo (MACV), Chapare, Guanarito, and Sabia viruses [5,6]. However, TCRV has not been associated with human illness; the sole anecdotal report refers to a laboratory-acquired event in the year 1976 associated with mild flu-like symptoms [7].

The mammarenaviruses genome is comprised of two negative-sense RNA segments named S (small) and L (large), each containing two open reading frames (ORF) in an ambisense orientation and separated by a non-coding intergenic region (IGR) [8]. The S segment encodes the nucleocapsid protein (NP) and the glycoprotein precursor (GPC), which is cleaved by cellular proteases to generate three subunits: the receptor-binding protein (GP1), the transmembrane fusion subunit (GP2), and a stable signal peptide (SSP). The three subunits remain noncovalently associated, forming the mature glycoprotein complex (GP) embedded in the viral envelope [9,10]. The L segment encodes the RNA-dependent RNA polymerase (L) and a RING matrix protein (Z) that is essential for viral morphogenesis. Multiple copies of NP associate with the L and S RNAs to form ribonucleocomplexes termed nucleocapsids, which in concert with the L polymerase function as transcription-replication units. Thus, NP and L are the minimal trans-acting viral factors required for viral RNA synthesis [11,12]. Genomic RNA segments contain non-coding regions (NCR) at their 5′ and 3′ termini, adjacent to the respective ORFs, which in the TCRV genome are 69 and 30 (L) and 68 and 76 (S) residues long, respectively. At the 3′ end, both the L and S segments contain a 19-nucleotide (nt) sequence highly conserved across arenaviruses and almost fully complementary to the corresponding 5′ terminal end, allowing for the formation of a panhandle structure [8,13,14]. For both genomic segments, the 5′- and 3′ terminal 19-nt sequences have been proven to promote genome transcription and replication [15,16,17]. The role of the terminal non-coding regions that separate these promoter sequences from viral ORFs has not been completely elucidated.

Current available evidence supports that NP and L mRNAs are first transcribed from the S and L genomic viral RNAs (vRNA). Transcription of GPC and Z mRNAs and vRNA replication requires synthesis and encapsidation of the S and L antigenomic RNAs (viral complementary, vcRNA) [18,19,20]. Genome replication by the L protein is thought to initiate *de novo* via a prime-and-realign mechanism, yielding a non-templated 5′ terminal G residue at both S and L genomes and antigenomes [21,22].

At the moment, there are no licensed vaccines to prevent disease caused by arenaviruses, except for the live attenuated JUNV vaccine (Candid#1), administered to adults (15 to 65 years old) at risk within AHF endemic areas in Argentina [23]. Candid#1 was developed using a conventional attenuation strategy starting from a human isolate [24]. Studies aimed at characterizing the Candid#1 strain in a murine model showed heterogeneity of the viral population in terms of virulence [25]. Genomic analysis revealed 13 amino acid changes scattered over NP, L, and GPC genes that differentiate Candid#1 from its parental strain XJ13 [26,27]. Studies employing recombinant Candid#1/XJ13 chimera viruses showed that a single amino acid mutation at the transmembrane domain of GP2 is decisive for the attenuated phenotype in a murine model [26], raising concerns on the risks of reversion of Candid#1 to virulence.

TCRV displays a close phylogenetic and antigenic relationship with JUNV. TCRV is apathogenic for guinea pigs and marmosets, in which JUNV infection reproduces human illness, and protects guinea pigs and non-human primates from lethal challenge with pathogenic strains of JUNV. Additionally, extensive attempts to induce TCRV-persistent infection in mice failed to induce chronic infections as those observed with JUNV [7,24]. Thus, based on these evidences and because the attenuated phenotype of TCRV is considered to be the result of a long evolutionary process, TCRV stands as a possible naturally live-attenuated virus vaccine candidate against AHF, with estimated low risks of changing into a pathogenic phenotype. Additionally, TCRV has been largely employed as a suitable model to study the biology of pathogenic NW mammarenaviruses in BSL2 laboratories [7,13,22]. 

Recombinant DNA technology enables engineering viral vectors that can be exploited as a platform to design new vaccines. Here, we report the development of a reverse genetic system to rescue recombinant TCRV (rTCRV). This system allowed us to generate chimeric recombinant TCRV expressing JUNV GPC, thus providing a valuable tool for recombinant vaccine design. Moreover, sequence substitution within the S RNA 5′ non-coding terminal region reduced rTCRV growth ability in BHK21 cells, while having little impact on viral growth in Vero cells. Our results suggest that controlled modification within the terminal non-coding sequences might be exploited to further improve the safety profile of rTCRV. Additionally, this reverse genetic system will deepen our knowledge of the molecular basis of viral attenuation.

## 2. Results

### 2.1. Generation of Infectious Recombinant TCRV

To establish a reverse genetic system to generate rTCRV, full-length antigenomic S and L RNA sequences were cloned under control of the bacteriophage T7 RNA polymerase (T7pol) promoter, obtaining plasmids pSag and pLag, respectively (see: Materials and Methods). Plasmid-driven intracellular synthesis of functional S and L antigenomes (Sag and Lag) in cells expressing T7pol was expected to be sufficient to trigger viral RNA transcription and replication, as documented for other segmented negative-sense RNA virus reverse genetic systems [28,29,30].

**Figure 1:**
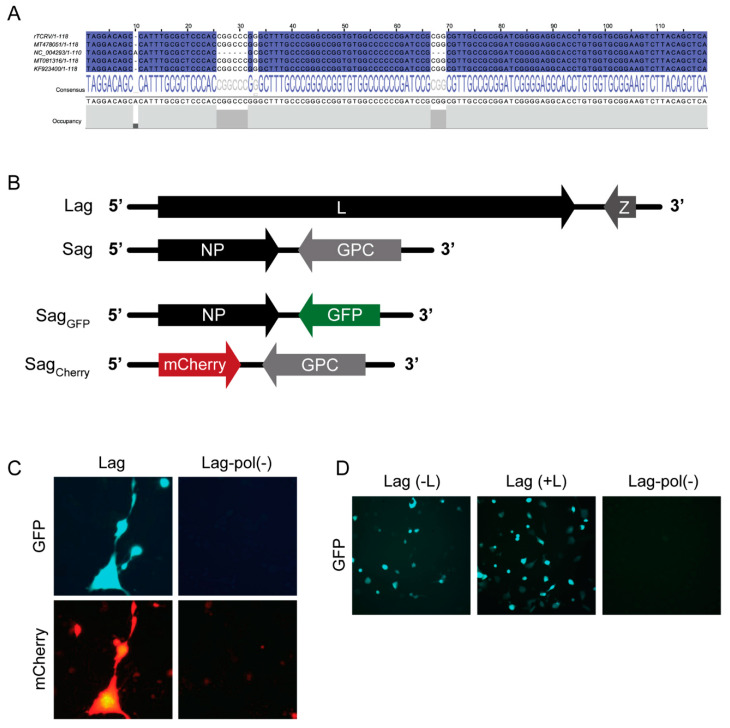
Generation of a reverse genetics system for rTCRV rescue. (**A**). T-Coffee alignment of the intergenic region (IGR) sequence (genomic sense) from the rTCRV S cDNA clone (rTCRV) and the indicated TCRV entries available from GenBank (Jalview Version: 2.11.1.2 [35]). Identical residues are marked in blue. (**B**). Schematic representation of S and L antigenomes (Sag and Lag, respectively), and T7pol-driven transcripts synthesized from pSagGFP (SagGFP) and pSagCherry (SagCherry). (**C**). Primary and secondary RNA transcription from S antigenome variants. BHK/T7-9 cells were transfected to express SagCherry, SagGFP and Lag or Lag-pol(−) as control, as indicated. The cells were observed under fluorescence microscope 48 h later. (**D**). Setting of transfection conditions by monitoring GFP expression. A representative experiment is shown. BHK/T7-9 cells were transfected with pSagGFP and pLag with (+L) or without (−L) the L-expressing plasmid pL. As control, parallel cultures were transfected with pSagGFP and pLag-pol(−). Samples were analyzed using fluorescence microscopy 48 h later.

Plasmids pLag and pSag were assembled on the basis of cDNA clones previously obtained, with sequences early reported under GenBank entries NC_004293 (S) and NC_004292 (L) [18,31,32]. Constructs were fully sequenced and given some discrepancies found within the S IGR as compared to the NC_004293 reference (Figure 1A), the TCRV S IGR was re-sequenced. We found 100% sequence identity between Sag IGR and all (eleven) individual cDNA clones obtained from TCRV-infected cell RNA using reverse transcription-polymerase chain reaction (RT-PCR) (data not shown). Furthermore, Sag and Lag shared over 99.7% overall sequence identity, including 100% identity in the S IGR (Figure 1A), with recently reported TCRV sequences [3,33,34]. Therefore, we concluded that differences found with the reference NC_004293 (obtained in the 1980s) can be explained by sequencing errors in that very early entry.

Next, to assess whether the S and L antigenome-expressing plasmids would be able to sustain viral RNA replication, we created a version of pSag that carried the green fluorescent protein (GFP) reporter gene in replacement of the GPC ORF (pSagGFP), as well as a version of pSag carrying the red fluorescent protein mCherry ORF in the NP locus (pSagCherry) (Figure 1B). Plasmids pSagCherry, pSagGFP, and pLag were co-transfected into BHK/T7-9, which constitutively expressed T7pol. As a control, parallel cultures were transfected with pSagCherry, pSagGFP, and pLag-pol(−), which coded for a catalytically inactive L polymerase (Materials and Methods). Expression of GFP and mCherry was monitored by fluorescence microscopy two days later. Both green and red fluorescence was detected in cells co-expressing the functional L polymerase and, as expected, no signal was visualized in cells transfected with pLag-pol(−) (Figure 1C). These results indicated that primary transcription of the reporter GFP gene from input SagGFP had taken place and, in addition, sufficient functional L and NP proteins were synthesized from input Lag and SagGFP RNAs, respectively, to support synthesis of the full-length genomic sense SgCherry RNA and subsequent transcription of Cherry mRNA. Therefore, we concluded that the system is fully functional and could support rTCRV rescue, without the need of additional helper plasmids to provide essential NP and L proteins that start viral RNA synthesis. Nevertheless, during various attempts to further increase GFP expression by transfecting BHK/T7-9 cells with variable amounts of pSagGFP and pLag, the addition of accessory NP or L-expressing plasmids was assayed. Thus, we found that inclusion of pL in the transfection mix resulted in three to four-fold increase of the number of fluorescent cells (Figure 1D). 

**Figure 2:**
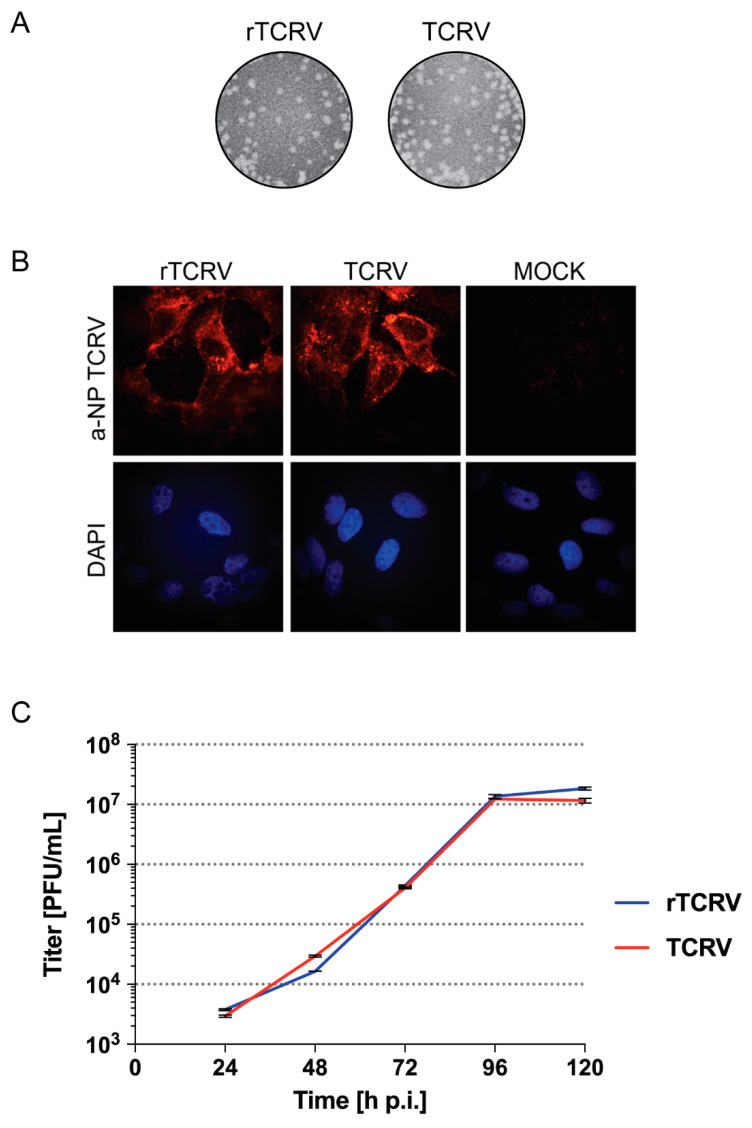
Rescue of rTCRV. (**A**). Recombinant and authentic TCRV plaque morphology. Vero cells were infected with working stocks of rTCRV or authentic TCRV, as indicated. At day 11 p.i., cells were fixed and viral plaques were visualized after standard staining of cell monolayers. (**B**). Detection of NP in Vero cells infected with the indicated virus was carried out by immunofluorescence, using an anti-TCRV NP polyclonal antibody. Mock, control uninfected cells. (**C**). Growth kinetics. Vero cells were infected by triplicate with rTCRV or authentic TCRV at MOI 0.005, as indicated in the Materials and Methods section. Aliquots of cell supernatants were collected at the indicated time points and the virus was titrated by standard plaque assay on Vero cells. Data correspond to the mean +/− standard deviation (SD).

Based on these results, subsequent rescue of infectious rTCRV was assayed by transfecting BHK/T7-9 cells with optimal amounts of plasmids pLag, pSag, and pL. Transfected cell supernatants (P0), analyzed after 3 or 6 day-incubation, presented titers of recovered rTCRV that consistently raised up to 10^3^ to 10^5^ plaque forming units (PFU) per milliliter. As expected, no virus could be detected in P0 when pLag and pL were omitted, and pLag-pol(−) was included instead in the transfection mix (data not shown). Moreover, rTCRV exhibited similar plaque morphology to that shown by authentic TCRV (Figure 2A), and TCRV NP was detected in cells infected with rTCRV, as determined by immunofluorescence (Figure 2B). Furthermore, in Vero cells, although rTCRV exhibited a moderate decreased growth at 48 h p.i., it still registered maximum viral titers at 96 h p.i. and reached final titers at 5 days p.i. similarly to authentic TCRV (Figure 2C).

### 2.2. rTCRV as a Tool for Recombinant Chimeric Virus Generation

**Figure 3:**
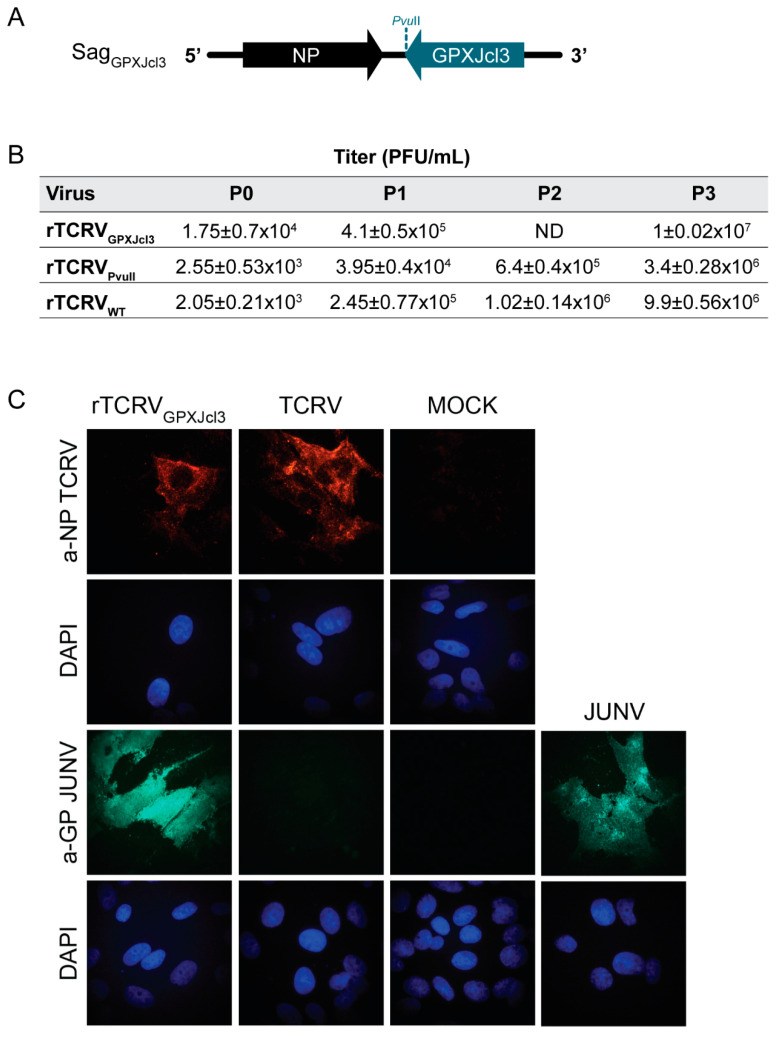
Rescue of a chimeric rTCRV expressing the JUNV glycoproteins. (**A**). Schematic representation of the chimeric TCRV/JUNV S antigenome Sag_GPXJcl3_. (**B**). Chart showing viral titers determined for the indicated recombinant virus passages. BHK/T7-9 cells were transfected with pL, pLag, and either pSag_GPXJcl3_, pSag_PVUII_ or pSag. Three days later, cell supernatants were collected (P0) and used to infect BHK21 cells. Culture supernatants collected at 3 days p.i., (P1) were employed to carry out two sequential passages (P2, P3) following the same methodology. All culture supernatants were collected at 3 days p.i. and mean viral titers (+/−SD) were determined by standard plaque assay on Vero cells. rTCRV_WT_, wild-type rTCRV. ND, not determined. (**C**). Detection of viral antigens by immunofluorescence. Vero cells were mock-infected or infected (MOI 1-3) with the indicated virus for 48 h. JUNV GP and TCRV NP were detected by immunofluorescence, using monoclonal anti-JUNV GP1 and polyclonal anti-TCRV NP, respectively, as a primary antibody (Materials and Methods). Nuclei were stained with DAPI.

We have previously reported the generation of infectious chimeric TCRV/JUNV viral-like particles from plasmid-transfected cells [36]. Moreover, rescue of recombinant viruses expressing heterologous GP has already been documented for arenaviruses [37,38,39]. Therefore, we anticipated that the TCRV reverse genetic system could be conveniently exploited to generate chimeric viruses, as a starting point towards the development of a recombinant TCRV-based vaccine platform. To this aim, we first constructed a suitable pSag vector by site-directed mutagenesis to introduce a PvuII site downstream of the GPC stop codon. Next, pSag_PVUII_ was used as backbone to create a chimeric pSag in which the GPC coding sequence from JUNV strain XJCl3 was placed in the TCRV GPC locus (pSag_GPXJcl3_, Figure 3A). Strain XJCl3 has been derived from the XJ prototype strain and it is highly attenuated in guinea pigs, being used early as an experimental human vaccine [23]. 

Using the experimental conditions set for rTCRV rescue, the chimeric rTCRV/JUNV was successfully recovered from BHK/T7-9 cells transfected with pL, pLag, and pSag_GPXJcl3_. As control, wild-type rTCRV and rTCRV_PVUII_ were rescued from culture monolayers transfected in parallel. Viruses present in transfected-cell supernatants (P0) were amplified by consecutive passages in BHK21 cells. Viral titers determined for each passage at 3 days p.i. demonstrated that the chimeric rTCRV_GPXJcl3_ grew at similar or even higher levels than those exhibited by either wild-type rTCRV or rTCRV**_PVUII_** (Figure 3B).

To further characterize the chimeric virus, Vero cells were infected with rTCRV_GPXJcl3_ passage 3 (P3), or with either TCRV or JUNV Candid#1 as a control. Infected monolayers were analyzed using immunofluorescence with an anti-JUNV GP1 specific Mab (BF-11) that did not cross-react against TCRV GP [40]. As a control, cells were probed with a monospecific polyclonal antiserum against TCRV NP. Expression of JUNV GP was clearly detected in cells infected with either rTCRV_GPXJcl3_ or JUNV, while no signal was observed in TCRV infected cells, as anticipated. Additionally, detection of TCRV NP in cell monolayers confirmed the infection with the chimeric virus and also validated the control infection with TCRV (Figure 3C). These results demonstrated that rTCRV_GPXJcl3_ readily expressed JUNV GPC and confirmed the feasibility of using our TCRV reverse genetic system as a platform for generating a TCRV/JUNV chimeric recombinant virus.

Studies employing a reverse genetic approach for the OW LASV have shown that recombinant viruses carrying deletion of the NCR sequence beyond the S RNA terminal 19-nt promoter presented an attenuated phenotype. Moreover, deletions in the S genome 5′ NCR were documented to have a stronger impact on virus growth efficiency than comparable deletions on the 3′ NCR [41]. Based on sequence comparison of Clade B mammarenavirus S RNA 5′ NCRs, two subregions stand out beyond the 19-nt terminal 5′ vRNA promoter. A proximal subregion displaying high identity, which corresponds to positions 20 to 35 from the 5′ end in TCRV S RNA, and a less conserved promoter-distal moiety comprising positions 36 to 68 (Supplementary Figure S1). 

**Figure 4:**
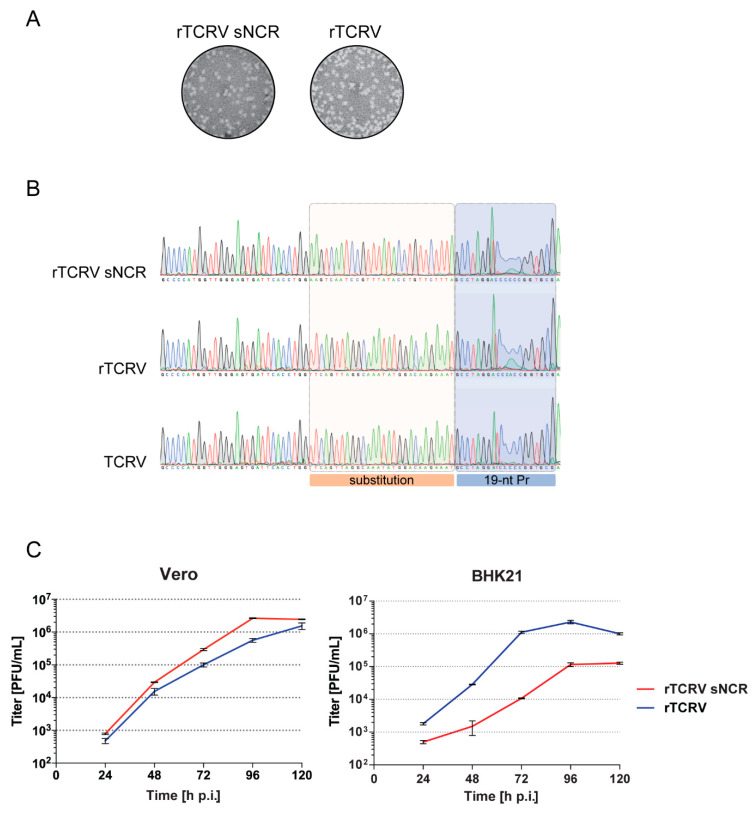
Rescue and characterization of mutant rTCRV_sNCR._ (**A**). Plaque morphology of mutant and wild-type recombinant viruses on Vero cell monolayers was determined as in Figure 2A. (**B**). Analysis of rTCRV S antigenome 3′ NCR sequence. Vero cells were infected with working stocks of mutant rTCRV_sNCR_ or, as control, with wild-type rTCRV or authentic TCRV. Total RNA purified from infected cells at 48 h p.i. was used to amplify the complete S vRNA 5′ NCR by RT-PCR, followed by sequencing. A section of the chromatograms including the 5′ vRNA promoter (19-nt Pr) and the 28-nt sequence changed in rTCRV_sNCR_, is shown. (**C**). Viral growth kinetics. Vero or BHK21 cells were infected by triplicate with mutant rTCRV_sNCR_ or wild-type rTCRV at MOI 0.005, as indicated in the Materials and Methods section. Aliquots of cell supernatants were collected daily and virus was titrated by standard plaque assay. Data correspond to the mean +/− SD.

Considering these observations, we asked whether the 5′ vRNA promoter-proximal non-coding subregion could play a role in viral multiplication. Thus, to start evaluating the role of terminal NCR in TCRV growth properties, we selected the sequence spanning positions 20 to 47 from the S 5′ end as target for mutagenesis and changed it by transversion in pSag, to generate mutant plasmid pSag_sNCR_. Using our reverse genetic approach, the mutant virus (rTCRV_sNCR_) was recovered in the supernatants of transfected BHK/T7-9 cells with titers about 10^4^ PFU/mL at day 6 post-transfection. Moreover, mutant rTCRV_sNCR_ formed plaques on Vero cells that were similar in size and morphology to those formed by wild-type rTCRV (Figure 4A).

In order to corroborate the genetic identity of the recovered viruses, total RNA was purified from Vero cells infected at MOI 4 with working stocks of mutant rTCRV_sNCR_ or rTCRV wild-type, and the S genome 5′ NCR (corresponding to 3′ NCR in S antigenome) was amplified and sequenced (Materials and Methods). As an additional control, parallel cultures were infected with authentic TCRV and processed in the same way. No sequence reversion was detected in rTCRV_sNCR_ in two independent experiments, indicating that the change introduced within the S genome 5′ non-coding region remained stable in the viral progeny up to passage 2 (Figure 4B). 

Next, we assessed the effect of the mutation within S 5′ NCR on viral growth using two different cell lines. In Vero cells, mutant rTCRV_sNCR_ displayed a slightly higher growth capacity as compared to wild-type rTCRV. Differences observed in viral titers at 72 and 96 h p.i. were statistically significant (*p* < 0.001), with both mutant and wild-type viruses reaching similar levels at 120 h p.i. (Figure 4C). In contrast, evaluation of viral growth properties in BHK21 cells revealed statistically significant lower titers of mutant rTCRV_sNCR_ than those of wild-type rTCRV at all-time points evaluated (*p* < 0.001). In addition, rTCRV_sNCR_ reached final titers at 5 days p.i that were 10 times lower than those observed for wild-type rTCRV (Figure 4C).

Overall, these results suggested that modification of the non-coding sequence neighboring the S 5′ vRNA promoter impacted on viral fitness in a cell type-dependent manner. 

## 3. Discussion

We documented here the development of a reverse genetic system that relies on the synthesis of full-length S and L antigenomic RNAs from T7 pol-driven plasmids, allowing us to successfully rescue infectious recombinant TCRV. As with a very recently reported system that depends on PolI/PolI-directed plasmids for rTCRV rescue [33], the T7 polymerase-based system presented here directs recovery of rTCRV, exhibiting in vitro phenotypical features close to those of authentic TCRV. 

Antibodies directed against envelope glycoproteins play an important role in protection against infection with JUNV and other New World arenaviruses [42,43,44,45,46]. Besides some evidence on cross-protection between MACV and Candid#1 *in vivo*, and studies on cross-neutralization of MACV with Candid#1 antibodies [24,47], there is no information supporting that the Candid#1 vaccine can be cross-protective against infection with other pathogenic mammarenaviruses. We employed the reverse genetic approach to generate a chimeric rTCRV that readily expresses GP from JUNV. Notably, the chimeric virus propagates in cell culture at similar levels than wild-type rTCRV (Figure 3). Considering our results and precedent studies reporting rescue of recombinant chimeric OW/NW arenaviruses [37,38], it is conceivable that the TCRV reverse genetic system presented here provides a useful alternative platform to generate chimeric viruses expressing GP from different NW, as well as OW pathogenic mammarenaviruses, potentially applicable to vaccine development. 

Using reverse genetic approaches, arenavirus genome non-coding sequences have been manipulated to obtain attenuated viral variants carrying deletions in the L IGR or substitution of the L IGR with the S IGR [33,48,49,50]. Regarding the 5′ and 3′ non-coding terminal sequences that separate the 19-nt promoter from the viral protein ORFs, evidence has been documented that although not being indispensable for propagation, the deletion of these regions impacts rLASV growth efficiency [41]. Accordingly, we were able to rescue mutant rTCRV_sNCR_ carrying substitution of a 28-nt sequence neighboring the 5′ vRNA promoter that is conserved across Clade B mammarenaviruses. Our observations suggest that the TCRV S 5′ NCR can tolerate changes without causing substantial impairment of virus rescue, as shown for LASV. Interestingly, although the mutant virus displayed similar plaque size in Vero cell cultures as compared with wild-type rTCRV, they exhibited dissimilar growth properties when evaluated in BHK21 cells. Indeed, mutant rTCRV_sNCR_ multiplicated at lower titers than wild-type rTCRV at all-time points (Figure 4), suggesting that the effect of substitution within the S 5′ NCR on viral fitness may depend on cell-type specific factor(s). These initial results pose the question of whether the S 5′ vRNA promoter-proximal non-coding sequence could harbor signal(s) involved in binding of cellular factors that may assist in viral RNA replication or encapsidation by NP. Additionally, in cell lines from phylogenetically distant host species (rodent and primate), pro-viral factors required to mediate viral life cycle could be expressed at different levels and/or display different affinities to viral RNA, therefore impacting on virus propagation. Indeed, several reports show evidence of distinct cell factors involved in arenavirus RNA synthesis [51]. Further work is required to explore whether genome NCRs could be evaluated as suitable targets for NW mammarenavirus attenuation *in vivo*. The reverse genetic system reported here will be useful to define additional attenuation markers that may contribute for the future design of safe mammarenavirus vaccine platforms. Likewise, it will also facilitate studies to precisely identify molecular determinants of NW arenaviruses pathogenesis, to explore on the molecular basis of viral replication dynamics and to further elucidate host factors implicated in virus multiplication.

## 4. Materials and Methods 

### 4.1. Cells and Viruses

African green monkey kidney cells (VERO 76, ATCC^®^ CRL-1587™) were maintained in Dulbecco’s modified Eagle’s medium-DMEM (Thermo Fisher, Waltham, MA, USA). Baby hamster kidney cells (BHK21 [C-13], ATCC^®^ CCL-10) and BHK/T7-9, stably expressing the T7 RNA polymerase [52], were grown in Glasgow minimum essential medium-GMEM (Thermo Fisher, Waltham, MA, USA). The growth medium was supplemented with 10% fetal bovine serum (FBS; Thermo Fisher, Waltham, MA, USA) and penicillin (100 U/mL)-streptomycin (100 μg/mL) (Thermo Fisher, Waltham, MA, USA). Hygromicin B (InvivoGen, San Diego, CA, USA) was added to a BHK/T7-9 culture medium in each of the 10 passages at a final concentration of 600 μg/mL, to select T7 polymerase expressing cells. 

JUNV Candid#1 was provided by Ricardo Gómez (Instituto de Biotecnología y Biología Molecular, CONICET-Universidad Nacional de La Plata, La Plata, Argentina). JUNV Candid#1 and TCRV (strain TRVL-11573) stocks were amplified in BHK21 cells. Viral titers were determined by plaque assay in Vero cells as described previously [53].

### 4.2. Plasmids 

Plasmids pL, expressing TCRV L protein under control of the T7 RNA polymerase promoter, was previously generated [36]. 

Plasmids pSag and pLag express the full-length antigenomic TCRV S and L RNAs, respectively, under control of the T7 polymerase promoter. Plasmids were constructed using a modified version of transcription vector pTV2.0 (pTV2.0 (Sac-)); transcripts were designed to contain an additional G at the 5′ end [11,54]. 

To obtain pLag, total RNA extracted from Vero cells infected for 96 h with TCRV at a multiplicity of infection (MOI) of 0.1 PFU/cell was used as a template to amplify the 5′ and 3′ regions of TCRV L genome using RT-PCR. To obtain the DNA fragment spanning positions 6087 to 7102 at the genome 3′ end, we used forward primer L3 fw and a reverse 19-nt oligonucleotide (L3 rv) designed on the basis of the L genome 3′ end sequence directly determined for JUNV and MACV. This sequence was previously demonstrated to be functional in reverse genetic systems [30,55]. The PCR product was phosphorylated and inserted at the StuI restriction site of pTV2.0(SacI-) immediately downstream of the T7 RNA polymerase promoter. The DNA fragment corresponding to the 5′ region (positions 1 to 193) of TCRV L RNA, obtained by using primers L5 fw and L5 rv, was phosphorylated and cloned at the SmaI site of the vector immediately upstream of the hepatitis delta virus ribozyme sequence (HDV Rz) that preceded a T7 polymerase terminator signal. The resulting plasmid was used as backbone to assemble the full length TCRV L antigenomic sequence by consecutive cloning of three different fragments, obtained from previously constructed plasmids pZ, p96 and pL [11]. 

pLag-pol(−) codes for a mutant L protein, which harbors a substitution within motif C that abrogates L polymerase activity. To construct pLag-pol(−), plasmid L C3 [56] was digested with BstEII, and the mutation (D1330A)-containing fragment was inserted into wild-type pLag digested with the same enzyme, in replacement of the wild type sequence. 

For the construction of pSag, the sequence comprised between positions 67 to 3066 of the TCRV S segment was obtained after digestion of clone p2b2 [18] with NcoI and BstEII and then was used to replace the NcoI-BstEII fragment in plasmid p15-2, a parental construct of pAgenCAT, which expressed a functional TCRV S antigenome analog [11]. In both plasmids pSag and pLag, an extra C nucleotide (nt) was inserted at the 3′ end of the antigenomic sequence by site-directed mutagenesis, using QuikChange PCR mutagenesis kit (Agilent, Santa Clara, CA, USA) and primers containing the mutation. This modification was reported to enhance the activity of the hepatitis delta virus ribozyme (HDV Rz) of the plasmid vector [16,57]. 

To generate plasmid pSagGFP, a DNA fragment comprised of the Enhanced Green Fluorescent Protein (EGFP) coding sequence was amplified by PCR from pEGFP-C3 (Takara Bio USA, Inc., Mountain View, CA, USA) using a phosphorylated 19-nt long reverse primer (GFP rv) corresponding to the 5′ terminus of GFP ORF, and a forward primer (GFP fw) including a SfiI site, 39 nt of the TCRV Sag IGR sequence and 23 nt complementary to the 3′-terminus of GFP ORF. The PCR product was cleaved with SfiI and the DNA fragment comprised of the GFP ORF was gel-purified. On the other hand, pSag was digested with NcoI followed by Klenow fill-in and then cleaved with SfiI. The vector-comprising fragment was then ligated to the GFP-encoding fragment to introduce GFP in the GPC locus.

Plasmid pSagCherry was obtained following a similar approach. Forward oligonucleotide (SacI-mCherry fw) was designed to contain the SacI restriction site sequence, followed by 30 nt of the S 5′antigenomic non-coding region (NCR) and the 5′ terminal 31-nt sequence of the mCherry red fluorescent protein ORF. The reverse primer (SfiI-mCherry rv) was comprised of the SfiI restriction site, followed by 60 nt of the S IGR sequence and a 27-nt sequence complementary to the 3′ terminal region of the mCherry ORF. These oligonucleotides were used to obtain a DNA fragment by PCR using pmCherry-N1 vector (Takara Bio USA, Inc., Mountain View, CA, USA) as a template. The PCR product was digested by SacI and SfiI restriction enzymes and the SacI-SfiI fragment was inserted between the same sites into the NP locus of pSag.

Plasmid pSag**_PVUII_** contains a PvuII site located 6 nt downstream of the GPC stop codon. It was obtained by introducing a two-nucleotide substitution into pSag, using QuikChange PCR mutagenesis kit (Agilent, Santa Clara, CA, USA) with primers pSagPvuII fw and pSagPvuII rv. The resultant mutant plasmid was excised with HpaI and SfiI and the DNA fragment carrying the PvuII site was swapped with the wild type HpaI-SfiI fragment in pSag.

To obtain plasmid pSag_GPXJcl3_, the coding sequence of GPC from JUNV strain XJCl3 was obtained by PCR using pcDNA3.1-GPC XJCl3 [58] as a template. Reverse primer (GPCjunv rv) was designed to contain a NcoI site and the 5′ terminal 18-nt sequence of the GPC JUNV XJCl3 ORF. Forward primer (PvuII-GPCjunv fw) was comprised of a PvuII site followed by a sequence that included 21 nt complementary to the 3′-terminal region of GPC JUNV XJCl3 ORF. The PCR product was digested with NcoI and PvuII restriction enzymes, the NcoI-PvuII fragment was purified and cloned between the same sites of pSag**_PVUII_**, to replace TCRV GPC with JUNV GPC.

To obtain mutant plasmid pSagsNCR, plasmid pSag was first digested with HindIII to avoid amplification of the antigenomic 5′ end, and then employed as a template to obtain a DNA fragment comprising the mutation by PCR. Forward primer (pSag-sNCR fw) contained a NcoI site and the Sag 3′ NCR sequence including the 28-nt substitution (sNCR) and the 19-nt 3′vcRNA promoter. Reverse primer (PstI-pSag-sNCR rv) corresponded to a 24-nt sequence of the vector covering a PstI unique site. The PCR product was cleaved with NcoI and PstI, and the NcoI-PstI fragment was cloned between the same sites of pSag, replacing the wild type fragment.

All plasmids were purified by using Qiagen Tip-100 (Qiagen Inc., Valencia, Spain). Construct sequences were confirmed by automated dideoxynucleotide DNA sequencing (Macrogen, Seoul, Korea). Complete rTCRV S and L sequences were deposed at GenBank with accession numbers MW071169 and MW071170, respectively. Primer sequences are shown in Supplementary Table S1. Vector maps are available upon request.

### 4.3. Rescue of Recombinant TCRV (rTCRV)

Optimal conditions for the generation of rTCRV were set as described in the text. Briefly, subconfluent monolayers of BHK/T7-9 cells, which constitutively express the T7 RNA polymerase, were transfected with (amounts per well of 6-well plates) 3.25 μg of pLag, 6 μg of pSag, and 0.1 μg of pL, using Lipofectamine 2000, according to the manufacturer’s instructions. The transfection medium was removed after 24 h-incubation and fresh medium supplemented with 1% FBS was added. Supernatants (P0) were collected at 3 or 6 days post-transfection, clarified by low-speed centrifugation and stored at −85 °C. Unless otherwise indicated, recombinant viruses were amplified in BHK21 cells after infection of cell monolayers with P0 at MOI 0.005, to reduce production of defective interfering particles [59]. Supernatants were collected at day 7 p.i. and used as viral working stocks. 

### 4.4. Viral Growth Curves

Subconfluent monolayers of Vero or BHK21 cells grown in 6-well plates were infected at MOI 0.005 for 1 h at 37 °C. Then, the inoculum was removed and fresh media supplemented with 1% FBS was added to each well. Aliquots of cell culture supernatants were collected at 1-day interval from 1 to 5 days p.i., clarified by centrifugation and titered by standard plaque assay on Vero cells.

### 4.5. Purification and Analysis of Viral RNA 

Infected-cell monolayers were lysed at the indicated times with TRIzol Reagent (Thermo Fisher, Waltham, MA, USA) and total RNA was extracted according to the manufacturer’s instructions. Purified total RNA was subjected to RT-PCR to obtain DNA fragments comprising the S genome target regions. Direct sequencing of a DNA fragment spanning positions 1 to 332 from the 5′ end (to control the identity of mutant rTCRV_sNCR_), or sequencing of individual cDNA molecules previously cloned into pGEM-T easy (Promega, Madison, WI, USA), covering positions 732 to 1748 (to analyzed S IGR), was performed by Macrogen Inc (Seoul, Korea). Oligonucleotides sequences are available upon request.

### 4.6. Immunofluorescence Microscopy

Vero cells grown on glass coverslips within 24-well dishes were infected with the indicated virus at MOI 1-3. At 48 h p.i. cells were washed twice with 1X PBS and fixed with 4% paraformaldehyde for 10 min at room temperature. After washing with PBS, cells were permeabilized by incubation with 0.2% Triton X-100 for 10 min at room temperature, blocked with PBS containing 3% bovine serum albumin (BSA) for 1 h, and incubated with the primary antibody in PBS containing 3% BSA, for 1 h at 37 °C. Antibodies used were: rabbit polyclonal anti-TCRV NP [60] or Mab QC03-BF11 directed against the GP1 subunit of JUNV GP [40], obtained through the NIH Biodefense and Emerging Infections Research Resources Repository, National Institute of Allergy and Infectious Diseases, NIH. After being extensively washed in PBS–3% BSA cells were incubated with the secondary antibody, for 1 h at 37 °C. Alexa Fluor 488 chicken anti-mouse immunoglobulin G (Thermo Fisher, Waltham, MA, USA) was used to detect JUNV GP and Alexa Fluor 568 goat anti-rabbit immunoglobulin G (Thermo Fisher, Waltham, MA, USA) was employed to detect TCRV NP. Following washing as indicated above, nuclei were stained with DAPI (Sigma-Aldrich, St Louis, MO, USA). Cells were mounted on FluorSave (Calbiochem, San Diego, CA, USA). Images were acquired with a Nikon Eclipse E600 microscope equipped with a Nikon DS-Fi1 camera.

### 4.7. Statistical Analysis

Statistical analyses were performed using the SPSS 17.0 statistical software package (SPSS, Inc., Chicago, IL, USA). The statistical significance between two groups was analyzed using Independent Samples T-Test.

## Supplementary Materials

The following are available online at https://www.mdpi.com/2076-0817/9/11/948/s1, Figure S1: Alignment of S RNA 5′ non-coding sequence from Clade B NW mammarenaviruses, performed with T-Coffee (Jalview Version: 2.11.1.2 [35]), Table S1: Oligonucleotides employed in cloning procedures.
