# Supplementary material for: Development of a Reverse Genetic System to Generate Recombinant Chimeric Tacaribe Virus that Expresses Junín Virus Glycoproteins
Source: Pathogens. 2020 Nov 13;9(11):948. doi: 10.3390/pathogens9110948 (PMC7696886; doi:10.3390/pathogens9110948)

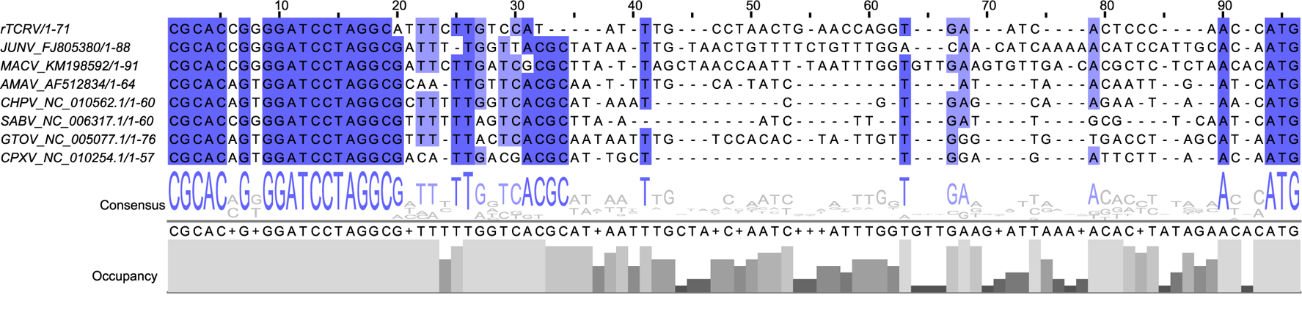


**Supplementary Figure 1.** Alignment of S RNA 5’ non-coding sequence from Clade B NW mammarenaviruses, performed with T-Coffee (Jalview Version: 2.11.1.2 [35]). The GPC start codon was included in all sequences. In blue, positions displaying over 75% identity. Genbank accession numbers are indicated for each virus. AMAV, Amapari; CHPV, Chapare; SABV Sabia; GTOV, Guanarito; CPXV, Cupixi. rTCRV, corresponds to the cDNA sequence determined in this work.

**Supplementary Table 1.** Oligonucleotides employed in cloning procedures. Restriction site sequences are underlined. Substitute sequence in pSag-sNCR fw is denoted in upper case characters.


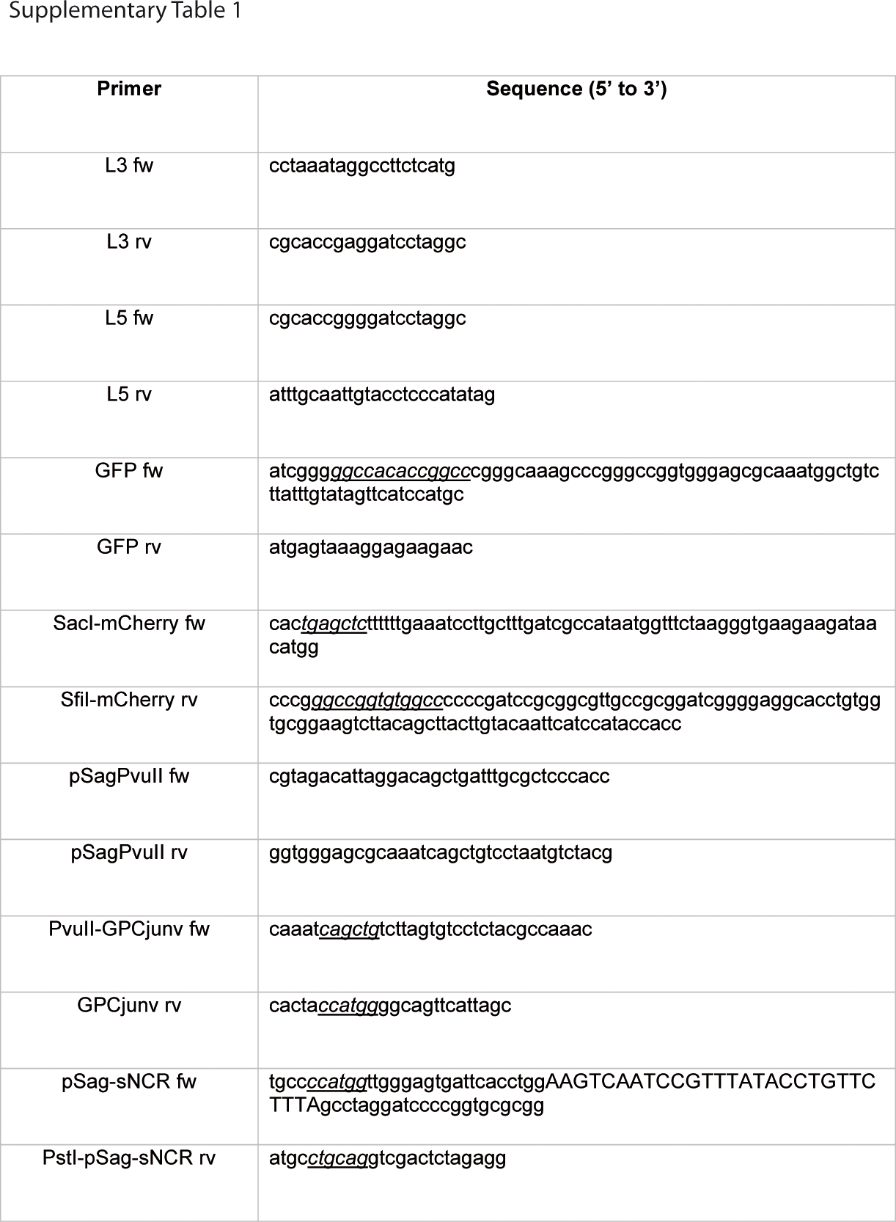

Supplement: Supplementary file 1 [file pathogens-09-00948-s001.zip › pathogens-975580-supplementary revised.docx]
