# Supplementary material for: Development of a Reverse Genetic System to Generate Recombinant Chimeric Tacaribe Virus that Expresses Junín Virus Glycoproteins
Source: Pathogens. 2020 Nov 13;9(11):948. doi: 10.3390/pathogens9110948 (PMC7696886; doi:10.3390/pathogens9110948)
Supplement: Supplementary file 1 [file pathogens-09-00948-s001.zip › Suppl Table 1 Foscaldi et al.docx]

Supplementary Table 1. Oligonucleotides employed in cloning procedures

| **Primer** | **Sequence (5’ to 3’)** |
| --- | --- |
| L3 fw | cctaaataggccttctcatg |
| L3 rv | cgcaccgaggatcctaggc |
| L5 fw | cgcaccggggatcctaggc |
| L5 rv | atttgcaattgtacctcccatatag |
| GFP fw | atcggg*ggccacaccggcc*cgggcaaagcccgggccggtgggagcgcaaatggctgtcttatttgtatagttcatccatgc |
| GFP rv | atgagtaaaggagaagaac |
| SacI-mCherry fw | cac*tgagctc*ttttttgaaatccttgctttgatcgccataatggtttctaagggtgaagaagataacatgg |
| SfiI-mCherry rv | cccg*ggccggtgtggcc*ccccgatccgcggcgttgccgcggatcggggaggcacctgtggtgcggaagtcttacagcttacttgtacaattcatccataccacc |
| pSagPvuII fw | cgtagacattaggacagctgatttgcgctcccacc |
| pSagPvuII rv | ggtgggagcgcaaatcagctgtcctaatgtctacg |
| PvuII-GPCjunv fw | caaat*cagctg*tcttagtgtcctctacgccaaac |
| GPCjunv rv | cacta*ccatgg*ggcagttcattagc |
| pSag-sNCR fw | tgcc*ccatgg*ttgggagtgattcacctggAAGTCAATCCGTTTATACCTGTTCTTTAgcctaggatccccggtgcgcgg |
| PstI-pSag-sNCR rv | atgc*ctgcag*gtcgactctagagg |
